# Comparing the efficacy of trabeculectomy and XEN gel microstent implantation for the treatment of primary open-angle glaucoma: a retrospective monocentric comparative cohort study

**DOI:** 10.1038/s41598-020-76551-y

**Published:** 2020-11-09

**Authors:** Theresa Theilig, Matus Rehak, Catharina Busch, Caroline Bormann, Marc Schargus, Jan Darius Unterlauft

**Affiliations:** 1 Department of Ophthalmology, University Clinic Leipzig, University of Leipzig, Liebigstrasse 10-14, 04103 Leipzig, Germany; 2 Department of Ophthalmology, Asklepios Clinic Heidberg, Tangstedter Landstrasse 400, 22417 Hamburg, Germany; 3 Universitäts-Augenklinik Düsseldorf, Universitätsklinikum Düsseldorf, Moorenstraße 5, 40225 Düsseldorf, Germany

**Keywords:** Eye diseases, Medical research, Outcomes research

## Abstract

The aim of this study was to compare the efficacy and safety profile of XEN microstent implantation with trabeculectomy (TET) in a comparable group of open-angle glaucoma cases in a retrospective, monocentric, single-surgeon setting. Each treatment group consisted of 100 eyes of 100 patients. At regular follow-up visits during the first 12 months after surgery, the following assessments were conducted and compared: intraocular pressure (IOP), number of IOP-lowering medications applied, best-corrected visual acuity (BCVA) and visual field testing. In both groups mean IOP was significantly reduced (p < 0.001). Mean IOP dropped from 24.8 ± 7.8 to 14.8 ± 4.0 mmHg in the TET and from 24.5 ± 6.7 to 16.6 ± 4.8 mmHg in the XEN group. The number of active compounds in the prescribed medication dropped from 3.3 ± 1.2 to 1.3 ± 1.4 in the TET and from 3.0 ± 1.1 to 1.4 ± 1.5 in the XEN group. BCVA and mean defect of static automated perimetry did not show a change of statistical significance in either group. Complications were more frequent after TET (p = 0.005) while postoperative needling was more frequent in the XEN group (p = 0.021). TET and XEN led to a significant reduction of IOP and IOP-lowering medication, while BCVA and visual field indices remained mostly unaltered over a 12-month postsurgical follow-up.

## Introduction

Glaucoma is after cataract the second leading cause of blindness worldwide with an estimated prevalence of 3–4% in the 40 years and older age group^1–3^. This estimate leads to presumably 76.0 million people affected by glaucoma in 2020^4^. Until 2040 a further increase to 111.8 million people is expected^5,6^. In glaucoma, apoptotic retinal ganglion cell demise leads to visual field loss, decrease of visual acuity (VA) and blindness in the final stages of the disease^6^. The causes leading to glaucoma are to date not fully understood. Chronically elevated intraocular pressure (IOP) due to a growing outflow resistance in the trabecular meshwork seems to play a major role^7^. Lowering IOP by medical and/or surgical means is the only proven therapeutic option by which further disease progression can be decelerated or stopped^8,9^.

Trabeculectomy (TET) was first described by Cairns more than 50 years ago and is the gold standard for the surgical treatment of glaucoma^10,11^. IOP is lowered through subconjunctival drainage of aqueous humor leaving production in the ciliary epithelium unaltered. Efficacy and availability of TET have been unrivaled for many decades, but the learning curve is comparatively shallow^11^. Minimally invasive glaucoma surgery (MIGS) techniques have been developed primarily for the treatment of primary open-angle glaucoma (POAG)^12^. The main reasons for the development of MIGS were to shorten operating time, decrease frequency and severity of complications, lower postoperative discomfort and to shorten convalescence times while lowering IOP effectively.

The XEN gelatin microstent (XEN 45 Gel Stent, Allergan, Irvine, California, USA) functions through the subconjunctival drainage of aqueous humor from the eye’s anterior chamber forming a prominent conjunctival filtration zone. The XEN microstent is a gelatin tube of 6 mm length and 500 μm diameter with an internal lumen of 45 μm. Due to its miniature size, flexible structure and the ab interno approach the surgical procedure implanting the XEN microstent seems to be less invasive and requires shorter operating time than standard TET, or implantation of other (bigger) drainage implants (Ahmed, Baerveldt, etc.)^13^. The IOP-lowering and medication-sparing efficacy of the XEN microstent has already been demonstrated in different single and multicenter trials^12,14–16^. The aim of this study was to compare the efficacy and safety profile of the XEN microstent with standard TET in a comparable group of POAG cases in a retrospective, monocentric single surgeon setting.

## Materials and methods

For this monocentric, retrospective, comparative cohort study all cases undergoing TET or XEN microstent implantation from October 2018 to July 2019 at the Department of Ophthalmology of the University Clinic Leipzig, Germany, were reviewed and included when meeting inclusion criteria. The required sample size for a non-parametric, dependent, two-sided test was calculated with an effect size of 0.3, a significance level (α) of 0.05 and a power of 0.8, resulting in a necessary sample size of 90 per group. 100 consecutive cases were retrospectively included in each treatment group, starting on the 1st of October 2018, with the last case being included undergoing surgery in July 2019. Standard procedure required every patient to present regularly for follow-up examinations during the first 12 months after surgery. The study was approved by the local ethics committee (209/18-ek). Written informed consent for all performed surgical procedures was obtained from all patients. All procedures performed involving human participants were conducted in accordance with the ethical standards of the institutional research committee and with the Declaration of Helsinki^17^. The performed clinical trial was registered with the German Clinical Trial Register (DRKS, trial number: DRKS00020800) which is part of the WHO registry network. All surgical interventions were performed by the same ophthalmic surgeon (JDU).

Diagnosis of POAG was based on the presence of typical glaucomatous optic disc changes in patients aged 40 years and above. Untreated IOP had to be 21 mmHg or above. TET or XEN microstent implantation was performed in cases presenting with disease progression under maximum tolerable medical treatment. Disease progression was verified using repeated visual field (VF) testing. For this at least three consecutive VF test results had to be available performed during the last 12 months indicating an increase of at least 2.0 dB of mean deviation during this period. Exclusion criteria were a patient age of < 40 years or a present glaucoma entity other than POAG. If both eyes of a single patient had to undergo surgery, only the results obtained from the first eye were included for analysis.

The indication whether to perform TET or XEN microstent implantation was defined when the XEN microstent was first introduced to our clinic, which was before the actual retrospective study was designed. The indications relied on the following assumptions: The XEN microstent was developed as a minimally invasive procedure with the goal to generate comparable postoperative results to those after TET in terms of IOP reduction^18–20^. The XEN microstent is implanted using an ab interno approach. Hence, a risk for causing inadvertent damage to lens and iris with following accelerated opacification of the crystalline lens exists during implantation of the XEN microstent. Additionally, TETs are presumed to produce more pronounced IOP reduction^21–23^. Therefore, TET was performed in eyes requiring more aggressive IOP reduction, such as only eyes, eyes with a clear crystalline lens and eyes in need for a complete stop of all topical IOP-lowering medication after surgery. The XEN microstent was implanted in all other (low-risk) cases included into this study.

XEN microstent implantation was combined with cataract extraction and intraocular lens (IOL) implantation when BCVA was > 0.2 logMAR due to a cloudy crystalline lens. TET was performed in cases with a present clear crystalline lens, cases with known allergies to most if not all IOP-lowering medication and all (functionally) monocular cases. Prior glaucoma surgery was no exclusion criterion but had to date back at least 1 year before planned TET or XEN microstent implantation. In cases of prior TET before XEN implantation TET had to date back at least 1 year, was only allowed to be performed once and prior slit lamp examination had to show no sign of a functioning filtering bleb and only minimal conjunctival scarring.

Before surgery each patient underwent a complete ophthalmological examination. This included taking the patient´s medical history, best corrected visual acuity (BCVA) testing with a phoropter (no pinhole) using Snellen charts (transformed to logMAR for statistical analysis), measuring the eyes objective refraction, slit lamp examination of the anterior and posterior segments with evaluation of the optic nerve head by indirect ophthalmoscopy and peripapillary oriented optical coherence tomography scan (OCT; Spectralis, Heidelberg Engineering, Heidelberg, Germany), static automated perimetry testing (Twinfield 2, Oculus, Wetzlar, Germany; 24–2 test strategy, 55 target points), and Goldmann applanation tonometry.

Patient demographics collected included: age, gender, laterality and surgical history of the operated eye. Other data analyzed during follow-up were IOP, BCVA, mean deviation (MD) of static automated perimetry and number of administered IOP-lowering eye drops and the included active compounds necessary to meet target IOP. These data were collected at seven time points: last day before surgery (pre-surgery), two days, 1 month, and 3, 6, 9 and 12 months after surgery. Additionally, at each follow-up visit, the anterior and posterior eye segments were evaluated for adverse events (conjunctival scarring, flat bleb, shallow anterior chamber, choroidal detachment, hypotony maculopathy, etc.). Visual field testing was performed at baseline and additionally at the visits 6 and 12 months after surgery. Apart from this, number and timing of additional necessary surgical interventions after XEN or TET were analyzed. This included laser suture lysis in cases of TET, needling and open revision procedures in cases of TET or XEN.

### Surgical techniques

#### TET with mitomycin C (MMC)

Ocular surface was disinfected using povidone iodine. Conjunctiva was opened at the limbus (fornix based). Conjunctiva and Tenon’s capsule were then elevated from the sclera creating a small pocket. Bleeding vessels were coagulated. A 3 × 3 mm sponge soaked with MMC (concentration 0.2 mg/mL) was inserted into the conjunctival pocket and left there for 2 min. Afterwards the sponge was removed and the scleral bed and conjunctival pocket were meticulously rinsed with 20 mL of Balanced Salt Solution. A scleral flap was then created approximately 4 × 4 mm in size with a thickness aimed at ½ to ¾ of the sclera. The eye’s anterior chamber was then entered underneath the scleral flap using a diamond knife aiming anterior to the scleral spur. Next, an iridectomy was created using forceps and scissors. The scleral flap was then re-approximated using 2–4 non-absorbable single button sutures. Number of sutures depended on the resulting IOP and visibility of aqueous humor protruding underneath the scleral flap. Finally, the conjunctiva was re-approximated to the limbus using 2–4 absorbable single button sutures to assure water tightness of the resulting bleb. During the postoperative course suturolysis or additional subconjunctival injection of 5-Fluorouracil (5-FU) was performed when needed according to the intraocular pressure and the visible function of the bleb.

#### XEN with mitomycin C (MMC)

Ocular surface was disinfected using povidone iodine. Two side port paracenteses were made, and the eye’s anterior chamber was filled with a dispersive viscoelastic agent. In the superior nasal quadrant, a mark 3 mm posterior to the limbus was made, where later the XEN implant is designed to pierce through the sclera. Then 0.1 mL or less MMC (concentration 0.1 mg/mL) was administered through the conjunctiva and into the Tenon’s capsule to form a bleb. The XEN microstent implant was inserted into the eyes anterior chamber mounted in its applicator. The applicator’s sharp tip was then aimed at the chamber angle and pierced through the chamber angle anterior to the trabecular meshwork and then directed through the adjacent sclera to form a 3 mm intrascleral track with an outer orifice 3 mm behind the limbus underneath the conjunctiva. The applicator’s internal mechanism then allowed the XEN microstent to be left in the formed intrascleral track with 1 mm of the microstent in the anterior chamber, 3 mm in the intrascleral track and 2 mm underneath the conjunctiva. The stent’s outer portion was then examined and freed using a 30-Gauge needle if trapped in Tenon’s capsule. Cases of combined XEN implantation and cataract surgery were performed using an additional 2.4 mm corneal tunnel incision at the 12 o’clock position before the above described implantation procedure of the XEN microstent took place.

The postoperative treatment regimen was similar in both groups. This included topical antibiotic (gentamicin; QID for 1 week), topical steroid (prednisolone acetate 1%; QID for 4 weeks and titrated thereafter based on clinical assessment of postoperative inflammation) and cycloplegics (atropine 0.5%; BID for 1 week). If postoperative needling was considered necessary decision was based on clinical assessment taking into consideration postoperative course of IOP and appearance of the conjunctival filtration zone. The needling procedure was considered as part of the standard postoperative care to improve aqueous flow and lower IOP in both treatment groups. Needling was routinely performed in a sterile fashion under the operating microscope using a 30G cannula to free and mobilize the XEN microstent from attachments of conjunctiva and Tenon and to open Tenon´s capsule in cases of TET. At the end of the needling procedure 0.1 mL of 5-Fluorouracil (concentration 50 mg/mL) was injected. Open revision of XEN or TET comprised (re-)opening the conjunctiva in a fornix based fashion and treating either increased fistulation (in cases of TET) with additional sutures or decreased fistulation due to conjunctival scar formation unresponsive to preceding needling procedures (in cases of TET or XEN) by excision of scar tissue and adhesions and re-suturing of the conjunctiva. Laser suture lysis and 5-FU treatment were performed in cases of TET when considered necessary based on clinical assessment and IOP development but did not follow a strict clinical regimen. Furthermore laser suture lysis and 5-FU treatment were not performed later than 12 weeks after surgery since postoperative healing and scar formation was considered to be completed by that time.

Clinical success was defined following the recommendations described in the Guidelines on Design and Reporting of Glaucoma Surgical Trials published by the World Glaucoma Association (WGA). For achieving complete clinical success IOP had to be lowered ≥ 20% compared to baseline before surgery without the additional use of IOP-lowering medication. For cases to be considered as complete or qualified success no further surgical procedure (cyclodestructive procedures, ALT, all other incisional glaucoma surgery) apart from laser suture lysis in TET cases, or needling procedures in TET and XEN cases was allowed. For achieving qualified clinical success IOP had to be lowered by ≥ 20% with the additional use of IOP-lowering medication when pre-surgical number of IOP-lowering agents was not exceeded. All cases not meeting these criteria and especially those cases which needed further incisional surgery to control IOP were considered as failures.

Electronic data capture and statistical analyses were performed using SAS software, Version 9.4, SAS System (SAS Institute Inc., Cary, NC, USA) and were performed by the authors JDU and MS. Patient characteristics such as age, BCVA, refraction, and visual field indices are given as mean and standard deviation. Data were tested and not normally distributed (Kolmogorov–Smirnov test for normal distribution: P < 0.05). Therefore, the following non-parametric tests were used for *within*-group comparisons and *between*-group comparisons: Wilcoxon signed-rank and Mann–Whitney U test, respectively. Chi-square and Fisher’s exact test for small frequencies were performed in frequency tables, with P < 0.05 indicating statistical significance.

### Ethical approval

The study was approved by the local ethics committee of the University Clinic of Leipzig, Germany (209/18-ek). All performed procedures were part of the routine care and in accordance with the ethical standards of the institutional and national research committee and with the 1964 Helsinki Declaration and its later amendments.

### Consent to participate

Informed consent for all surgical procedures was obtained from all individual participants included in the study.

## Results

In total 200 eyes of 200 patients were included. Each treatment group consisted of 100 eyes. All 200 eyes suffered from POAG. A full follow-up existed for all 200 eyes. Patient demographics of both treatment groups were comparable and well balanced (Table 1). The percentage of eyes previously undergoing glaucoma surgery was 41% in the TET group and 44% in the XEN group. In the TET group 67 surgical IOP-lowering procedures were performed before TET. These were 38 argon laser trabeculoplasties (ALT) and 29 cyclophotocoagulations (CPC). In the XEN group 80 surgical IOP-lowering procedures were performed before XEN implantation. These were 38 ALT, 37 CPC and 5 TET. XEN microstent implantation was combined with phacoemulsification and IOL implantation in 52 eyes, the remaining 48 eyes were treated with XEN implantation as a stand-alone procedure. All TET cases were performed as a stand-alone procedure and none were combined with phacoemulsification and IOL placement.

**Table 1:** Demographics and baseline characteristics for the TET and XEN groups.

		TET	XEN	p-value
Gender	Females Males	56 44	52 48	0.67^§^
Age (years)	Mean (95% CI)	70.3 68.6|72.1	70.9 68.6|73.2	0.12^§§^
Laterality	Left eye Right eye	47.0% 53.0%	54.0% 46.0%	0.40^§^
Number of surgeries in preoperated eyes	0	59 (59%)	56 (56%)	0.57^§§^
	1	22 (22%)	22 (22%)	
	2	14 (14%)	14 (14%)	
	3	3 (3%)	3 (3%)	
	4	2 (2%)	4 (4%)	
	5	–	1 (1%)	

*CI* confidence interval, *TET* trabeculectomy group, *XEN* XEN microstent group.

^§^Fisher’s exact test.

^§§^Mann–Whitney U test.

**Figure 1:**
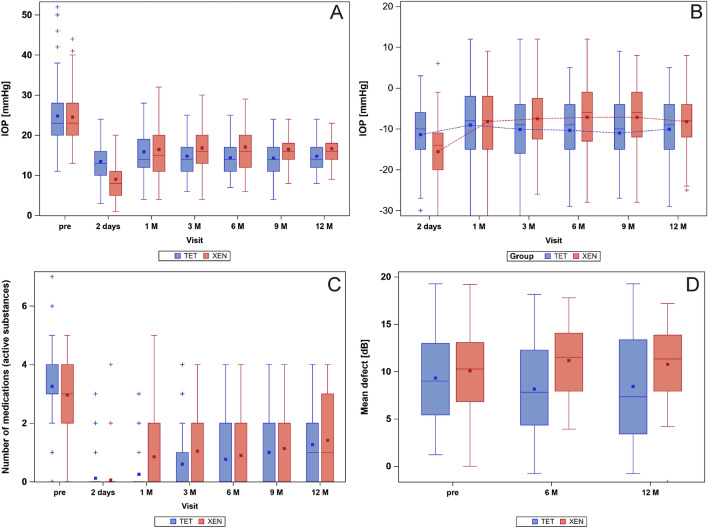
Box and whisker plots depicting (**A**) the course of mean IOP, (**B**) the course of mean IOP change compared to baseline values, (**C**) the mean number of applied active compounds of IOP-lowering agents and (**D**) the course of mean MD at baseline and during the first 12 months of follow-up after TET (blue) or XEN microstent implantation (red). *IOP* intraocular pressure, *M* months, *TET* Trabeculectomy group, *XEN* XEN microstent group, *2 days* day 2 after surgery, *1–12 M* follow-up visits from 1–12 months, *pre* baseline value before surgery.

**Figure 2:**
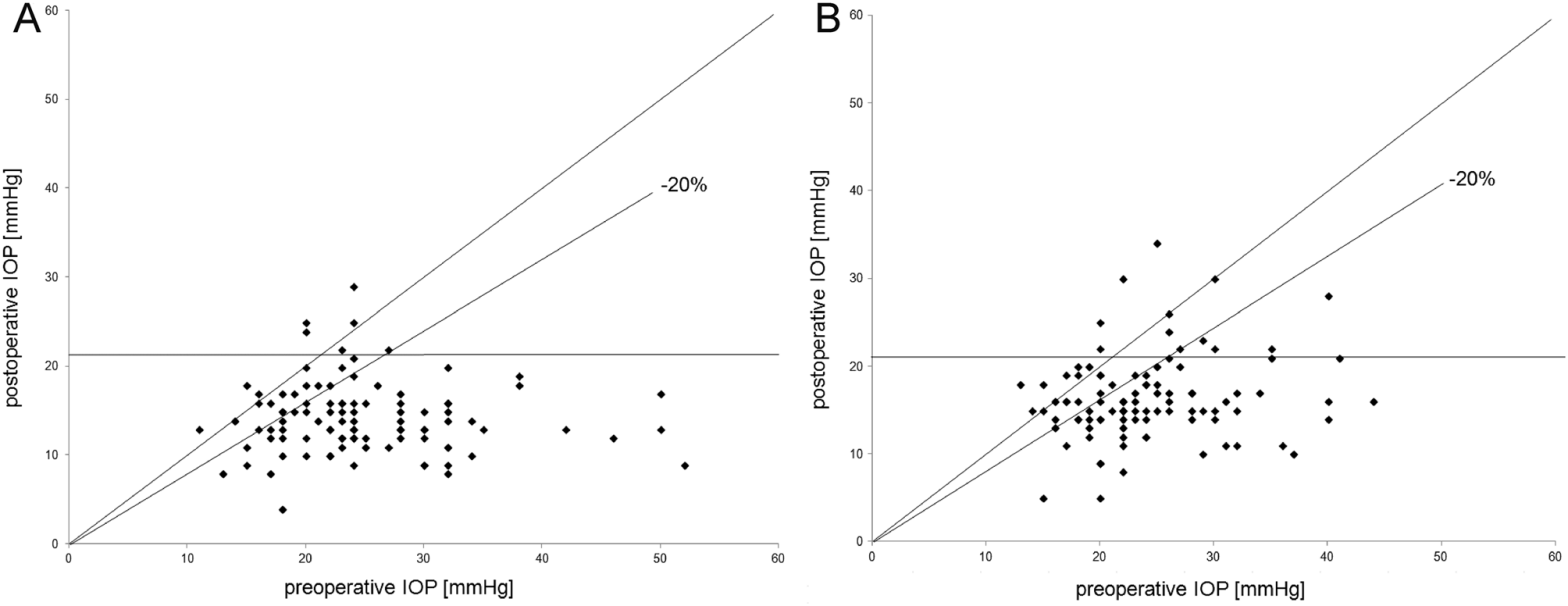
Scattergram comparing presurgical to 12 months postoperative IOP results for all cases operated in the TET (**A**) and XEN (**B**) groups. The − 20%-line indicates the level beneath which an IOP reduction of more than 20% compared to baseline value before surgery was reached by the individual cases. *IOP intraocular pressure).*

Before surgery mean IOP was similar and without statistical significant difference in both treatment groups (Mann–Whitney U test: p = 0.97). In the TET group mean IOP was 24.8 ± 7.8 mmHg before surgery. In the XEN group mean IOP was 24.5 ± 6.7 mmHg before surgery. After surgery mean IOP dropped effectively. Comparison of mean IOP values after surgery to results obtained at baseline before surgery were of statistical significance at each follow-up visit for both treatment groups (Wilcoxon signed-rank test: p < 0.001). At the 12-month follow-up visit mean IOP was 14.8 ± 4.0 mmHg in the TET and 16.6 ± 4.8 mmHg in the XEN group. Significant differences between the two groups were found for the 6-, 9- and 12-month visits (Mann–Whitney U test: p < 0.001). In the 52 cases of combined XEN and phacoemulsification surgery mean IOP dropped from 24.8 ± 6.9 mmHg before surgery to 16.4 ± 4.2 mmHg 12 months after surgery. In the 48 cases of XEN microstent implantation as a stand-alone procedure mean IOP respectively dropped from 24.4 ± 6.6 to 16.6 ± 5.9 mmHg. Comparing IOP results to measurements before surgery showed a difference of statistical significance at each follow-up visits (Wilcoxon signed-rank test: p < 0.001). Intergroup comparison between the combined and the stand-alone XEN cases showed a difference of statistical significance only at the follow-up visit at the second day after surgery (Mann–Whitney U test: p < 0.01). At all other visits IOP difference between combined and stand-alone XEN cases was not of statistical significance. The exact course of postsurgical IOP development for both groups can be taken from Fig. 1A,B and Tables 2 and 3. A Scattergram comparing IOP at baseline and 12 months after surgery for the XEN and the TET groups is depicted in Fig. 2.

**Table 2:** Course of mean IOP and mean number of IOP-lowering medications (active compounds) at baseline and during 12 months of post-surgical follow-up per group.

IOP [mmHg]				95% CI		p-value (Wilcoxon signed rank)	Number of active compounds		95% CI		p value (Wilcoxon signed-rank
Group	Visit	Mean ± SD		LL	UL		Mean ± SD		LL	UL	
TET	Pre-	24.8	7.8	23.3	26.4		3.26	1.23	3.01	3.50	
	2 days	13.4	5.1	12.4	14.5	< 0.001	0.12	0.48	0.03	0.21	< 0.001
	1 month	15.9	6.5	14.6	17.2	< 0.001	0.25	0.72	0.11	0.40	< 0.001
	3 months	14.8	5.4	13.7	15.9	< 0.001	0.60	1.12	0.37	0.83	< 0.001
	6 months	14.4	4.1	13.5	15.2	< 0.001	0.76	1.16	0.52	1.00	< 0.001
	9 months	14.3	4.7	13.3	15.3	< 0.001	1.00	1.29	0.72	1.28	< 0.001
	12 months	14.8	4.0	13.9	15.7	< 0.001	1.27	1.38	0.96	1.58	< 0.001
XEN	Pre-	24.5	6.7	23.2	25.9		2.96	1.13	2.74	3.18	
	2 days	9.0	5.6	7.9	10.2	< 0.001	0.06	0.45	-0.03	0.15	< 0.001
	1 month	16.5	8.2	14.8	18.1	< 0.001	0.85	1.44	0.55	1.15	< 0.001
	3 months	16.8	6.3	15.5	18.2	< 0.001	1.05	1.37	0.75	1.34	< 0.001
	6 months	17.1	7.7	15.3	18.8	< 0.001	0.90	1.36	0.59	1.20	< 0.001
	9 months	16.5	4.7	15.4	17.6	< 0.001	1.13	1.48	0.78	1.49	< 0.001
	12 months	16.6	4.8	15.3	17.9	< 0.001	1.42	1.50	1.00	1.83	< 0.001

*CI* confidence interval, *SD* standard deviation, *LL* lower limit, *UL* upper limit, *IOP* intraocular pressure, *TET* trabeculectomy group, *XEN* XEN microstent group, *pre* presurgical baseline value, *2 days* day 2 after surgery, *1–12 months* follow-up visits from 1 month–12 months.

**Table 3:** Course of mean IOP, mean number of IOP-lowering medications (active compounds) and BCVA at baseline and during 12 months of post-surgical follow-up for the XEN subgroups either treated with XEN microstent implantation combined with phacoemulsification and IOL implantation (XEN + phaco) or XEN microstent implantation as a stand-alone procedure (XEN alone).

XEN + phaco							XEN stand-alone					p value Mann–Whitney U test
				95% CI		p-value (Wilcoxon signed rank)			95% CI		p value (Wilcoxon Signed-Rank	
	Visit	Mean ± SD		LL	UL		Mean ± SD		LL	UL		
IOP	Pre-	24.8	6.9	22.3	26.7		24.4	6.6	22.2	26.3		0.81
	2 days	10.3	5.6	9.2	11.8	< 0.001	7.7	5.4	6.6	9.3	< 0.001	< 0.01
	1 month	15.8	6.9	13.8	17.7	< 0.001	16.1	9.4	13.8	18.7	< 0.001	0.54
	3 months	16.2	5.7	14.9	17.8	< 0.001	16.4	7.1	14.6	18.5	< 0.001	0.93
	6 months	16.4	5.3	14.9	17.8	< 0.001	16.1	8.0	13.9	18.4	< 0.001	0.44
	9 months	16.4	4.8	15.1	17.7	< 0.001	16.7	6.1	14.8	18.5	< 0.001	0.66
	12 months	16.4	4.2	15.5	17.6	< 0.001	16.6	5.9	14.7	18.3	< 0.001	0.93
Meds	Pre-	2.76	1.1	2.48	3.05		3.13	1.16	2.79	3.45		0.16
	2 days	0.04	0.28	-0.04	0.12	< 0.001	0.08	0.58	-0.08	0.25	< 0.001	0.97
	1 month	0.75	1.29	0.39	1.1	< 0.001	0.79	1.47	0.37	1.21	< 0.001	0.94
	3 months	0.88	1.27	0.53	1.23	< 0.001	1.0	1.46	0.58	1.42	< 0.001	0.72
	6 months	0.74	1.23	0.4	1.08	< 0.001	0.93	1.45	0.51	1.36	< 0.001	0.46
	9 months	0.88	1.35	0.5	1.26	< 0.001	1.16	1.46	0.73	1.58	< 0.001	0.29
	12 months	0.92	1.37	0.53	1.3	< 0.001	1.41	1.53	0.96	1.86	< 0.001	0.19
BCVA	Pre-	0.4	0.5	0.14	0.26		0.48	0.62	0.3	0.65		0.89
	2 days	0.44	0.54	0.28	0.59	0.42	0.58	0.61	0.41	0.76	0.01	0.02
	1 month	0.35	0.47	0.22	0.48	0.03	0.53	0.59	0.36	0.7	0.16	0.03
	3 months	0.26	0.41	0.14	0.38	< 0.01	0.49	0.6	0.32	0.67	0.34	< 0.01
	6 months	0.3	0.44	0.18	0.43	0.01	0.48	0.58	0.31	0.65	0.19	0.03
	9 months	0.28	0.44	0.15	0.41	0.02	0.46	0.56	0.29	0.63	0.13	0.01
	12 months	0.28	0.42	0.16	0.4	0.01	0.51	0.56	0.34	0.68	0.16	< 0.01

*CI* confidence interval, *SD* standard deviation, *LL* lower limit, *UL* upper limit, *IOP* intraocular pressure, *pre* presurgical baseline value, *2 days* day 2 after surgery, *1–12 months* follow-up visits from 1 month–12 months.

The mean number of active compounds applied was 3.3 ± 1.2 in the TET group and 3.0 ± 1.1 in the XEN group before surgery. Within each treatment group, the number of applied IOP-lowering agents at each follow-up visit compared to baseline were statistically significant (Wilcoxon signed-rank test: p < 0.001). A distinct drop of necessary IOP-lowering medication was detected for both combined and stand-alone XEN microstent implantation cases. The different numbers of applied IOP-lowering medications in the combined and stand-alone XEN cases was not of statistical significance. The exact course of the mean number of applied IOP-lowering compounds administered in each treatment group can be taken from Tables 2 and 3 and Fig. 1C.

12 months after surgery, 39% of eyes showed a complete and 74% a qualified success in the TET group. In the XEN group, 33% showed a complete and 67% qualified success 12 months after surgery (Table 4). In the subgroups treated with the XEN microstent complete success was achieved by 34% of the stand-alone cases and 32% of the combined cases. Qualified success was slightly more frequent in the combined cases (70%) than in the stand-alone cases (66%). 12 months after surgery the percentage of cases considered as failures for not reaching beforehand defined success definitions was 33% in the XEN group and 26% in the TET group. The different frequencies of complete and qualified success in the TET and XEN groups did not show statistical significance (see also Table 4).

**Table 4:** Rates of complete, qualified and no success reached at the follow-up visits 9 and 12 months after surgery for the groups treated with TET or XEN.

Visit	No success/failure		Qualified success		Complete success		p-value (Chi-Square)
	TET %	XEN %	TET %	XEN %	TET %	XEN %	
9 months	23	37	77	63	47	37	0.1775
12 months	26	33	74	67	39	33	0.6449

*TET* trabeculectomy group, *XEN* XEN microstent group, *9 & 12 months* follow-up visits 9 and 12 months after surgery.

Before surgery the difference of mean BCVA between XEN and TET groups was of statistical significance (Mann–Whitney U test: p < 0.0001). The difference remained so during the complete 12 months of follow-up. In the TET group worsening of mean BCVA from the second day after surgery onwards was statistically significant (Wilcoxon signed-rank test: p < 0.001). In the XEN group change of BCVA was not of statistical significance. However, mean BCVA improved in the subgroup treated with combined XEN microstent implantation and phacoemulsification. The difference of BCVA compared to baseline was of statistical significance from the follow-up visit 1 month after surgery onwards (Wilcoxon signed-rank test: p = 0.03 and below). In the subgroup treated with XEN microstent implantation alone mean BCVA remained stable during the first 12 months after surgery. The mean BCVA difference between both XEN subgroups was of statistical significance from the follow-up visit 2 days after surgery onwards (Mann–Whitney U test: p = 0.03 and below). The exact postsurgical course of mean BCVA can be taken from Tables 3 and 5.

**Table 5:** Pre- and postsurgical best corrected visual acuity (BCVA [logMAR]) and course of visual fields mean defect for TET and XEN microstent groups.

	Group	Visit	Mean ± SD		95% CI		p-value (Wilcoxon signed-rank)
					LL	UL	
Visual acuity [logMAR]	TET	Pre-	0.17	0.27	0.11	0.22	
		1 month	0.28	0.27	0.22	0.33	< 0.001
		3 months	0.26	0.31	0.20	0.32	< 0.001
		6 months	0.23	0.29	0.17	0.29	< 0.001
		9 months	0.21	0.22	0.16	0.25	< 0.001
		12 months	0.22	0.25	0.16	0.27	< 0.001
	XEN	Pre-	0.47	0.59	0.36	0.59	
		1 month	0.48	0.58	0.36	0.60	0.66
		3 months	0.40	0.56	0.28	0.52	0.07
		6 months	0.41	0.53	0.29	0.52	0.78
		9 months	0.43	0.55	0.30	0.56	0.93
		12 months	0.44	0.54	0.29	0.58	0.94
Mean defect [dB]	TET	Pre-	9.3	4.6	8.4	10.3	
		6 months	8.2	5.0	7.1	9.2	0.71
		12 months	8.4	5.5	7.1	9.7	0.23
	XEN	Pre-	10.1	4.3	9.2	11.0	
		6 months	11.2	3.8	10.2	12.2	0.24
		12 months	10.8	4.0	9.4	12.1	0.19

*CI* confidence interval, *SD* standard deviation, *LL* lower limit, *UL* upper limit, *IOP* intraocular pressure, *TET* trabeculectomy group, *XEN* XEN microstent group, *pre* presurgical baseline value, *2 days* day 2 after surgery, *1–12 months* follow-up visits from 1 month–12 months after surgery, *BCVA* best corrected visual acuity, *logMAR* logarithm of the minimum angle of resolution.

The mean defect (MD) of static automated perimetry was 9.3 ± 4.6 dB in the TET group and 10.1 ± 4.3 dB in the XEN group before surgery (Table 5 and Fig. 1D). This difference did not demonstrate statistical significance (Mann–Whitney U test: p = 0.21). At the 6-month and 12-month visits MD improved to 8.2 ± 5.0 dB and 8.4 ± 5.5 dB in the TET group. In the XEN group MD also worsened to 11.2 ± 3.8 dB and 10.8 ± 4.0 dB at the 6- and 12-month follow-up visits. However, 6- and 12-months results did neither in the TET (p = 0.71 and p = 0.23 respectively) nor in the XEN group (p = 0.24 and p = 0.19 respectively) show a difference of statistical significance when compared to baseline.

Temporary hypotony was noted 16% of eyes in the TET group and in 30% of eyes in the XEN group (i.e., IOP values ≤ 5 mmHg) during the first month after surgery. In all cases hypotony resolved without further sequelae. 9% of eyes in the TET group and 14% in the XEN group developed circumscribed choroidal detachment during the first month after surgery. Choroidal detachment also resolved without further necessity for surgical intervention in all cases. From the follow-up examination 1 month after surgery neither in the TET nor in the XEN group hypotony or choroidal detachment was detected. No case of hypotony induced maculopathy was detected. Postoperative hypotony was more common in the XEN group as compared to the TET group (Fisher’s exact test: p = 0.03). During the first 12 months of follow-up a needling procedure was necessary in 42% of eyes in the XEN group and 22% of eyes in the TET group (Table 6). This difference was of statistical significance (Fisher’s exact test: p = 0.02). Two needling procedures were necessary in 5% and 8% of eyes in the TET and XEN groups. More than two needling procedures were only necessary in 1 eye of the XEN group.

**Table 6:** Number of necessary needling procedures, type and frequency of occurring postsurgical complications/adverse events and type and frequency of necessary secondary interventions/postsurgical care measures in the TET and XEN microstent implantation groups during the first 12 months of follow-up after surgery.

	TET	XEN
**Needlings**		
0	78	58
1	17	33
2	5	8
3	0	1
**AE category**		
Bleeding in anterior chamber	8	7
Retinal detachment	1	0
Choroidal detachment (during first month after surgery)	9	14
Corneal erosion	5	0
Conjunctival bleeding	12	4
Hypotony (IOP < 5 mmHg) (during first month after surgery)	16	30
Other	3	4
	**54**	**59**
**Postsurgical care**		
Bleb revision	5	5
Resuturing	4	0
Suturolysis	21	0
TET revision	2	0
XEN revision	0	10
Other	1	1
	**33**	**16**

*N* number of times needling was necessary, *TET* trabeculectomy group, *XEN* XEN microstent group, *AE* adverse event.

During follow-up, several adverse events and complications were observed in both treatment groups. The encountered complications are summarized in detail in Table 6. The rate of adverse events was comparable in the TET- and XEN groups (Chi-square: p = 0.44). However, postsurgical complications requiring surgical attention (bleb revision, resuturing, laser suture lysis, TET revision, XEN revision etc.) were more frequent in the TET group than in the XEN group (33% vs 16%; Chi-square: p = 0.0403) (for details see Table 6).

## Discussion

This study demonstrates that IOP can be lowered effectively by both TET + MMC and XEN microstent implantation in a comparative manner with a statistical significant difference to baseline values. In both treatment groups mean number of necessary IOP-lowering medication could be reduced effectively. However, after 12 months of follow-up IOP values were lower in the TET than in the XEN group.

In our TET group mean IOP dropped from 24.8 ± 7.8 to 14.8 ± 4.0 mmHg 12 months after surgery, which represents a drop of 40.3% compared to baseline values. In the TET group complete and qualified success rates of 39% and 74% were achieved. Other groups found even more pronounced postoperative IOP decreases and larger success rates depending on varying success definitions applied^23–25^. In a multicenter trial (conducted in the UK) mean values of 12.4 ± 4.0 mmHg and success rates up to 87% were demonstrated 24 months after TET^23^. This might be explained with more aggressive treatment modalities utilized, although reported complication rates were comparatively low. Other than in our study, only eyes without prior glaucoma surgery were included and releasable scleral flap sutures were utilized. This may partly explain the lower mean IOP rates achieved compared to our hereby described results. Threshold criteria for commencing topical IOP-lowering medication after surgery to meet targeted pressure values may have been lower in our study population than in others described before. This may partly explain the large difference between complete and qualified success rates in our TET and XEN groups. Our results concerning our TET group seem more congruent with those found using a nationwide survey also conducted in the UK in the 1990s with a mean IOP of 14.4 mmHg and complete and qualified success rates of 67% and 71% 12 months after surgery^22,26^.

In our group of eyes treated with XEN microstent implantation mean IOP dropped from 24.5 ± 6.7 to 16.6 ± 4.8 mmHg and the mean number of applied IOP-lowering medications sank from 3.0 ± 1.1 to 1.4 ± 1.5 12 months after surgery. Reitsamer et al. demonstrated a similar decrease from 21.4 ± 3.6 mmHg on 2.7 ± 0.9 drops at baseline to 14.9 ± 4.5 mmHg on 0.9 ± 1.1 drops and 15.2 ± 4.2 mmHg on 1.1 ± 1.2 drops 12 and 24 months after XEN microstent implantation^27^. However, concerning these data it has to be noted that only eyes without prior incisional glaucoma surgery were included into this analysis and that a full follow-up existed only for 80% of eyes. Heidinger et al. described an IOP drop from 22.8 ± 6.9 mmHg on 2.9 ± 1.0 IOP-lowering medications to 17.1 ± 5.9 mmHg on 1.8 ± 1.4 IOP-lowering medications after 12 months of follow-up^28^. Pérez-Torregrosa et al. reported an IOP reduction of 29.3% from baseline in 30 eyes with combined phacoemulsification and XEN microstent implantation after 12 months of follow-up^29^.

Our results demonstrated a higher complete success rate in the TET group at the 12-month visit (complete success: TET: 39%; XEN: 33%; qualified success: TET: 74%; XEN: 67%). However, comparison of these rates did not show a difference of statistical significance. It can be argued that TET is more effective than XEN in terms of IOP reduction without medication, which would sustain its reputation of being the gold standard of filtering glaucoma surgery, but still both techniques seem sufficiently effective^30^. Similar results for the XEN microstent were reported from the APEX study with a clinical success rate of 67.6% at 12 months and 65.8% at 24 months after surgery^27^.

A higher prevalence for hypotony after XEN than after TET in the short-term postsurgical follow-up (as demonstrated by the low mean IOP of 9.0 ± 5.6 mmHg two days after surgery in the XEN group) has also been reported before^30^. However, in this present study, hypotony had resolved through conservative management in all cases during the first postoperative month without further sequelae. It has been argued before that postoperative hypotony by increased outflow of aqueous humor may even trigger a pronounced conjunctival fibrosis and therefore increase the rate of needling procedures^31,32^.

Regarding BCVA, our data demonstrate a significant decrease in the TET group and no measurable change in the XEN group 12 months after surgery. This was also the case when the effect of a further opacification of the crystalline lens was ruled out and only results obtained from already pseudophakic eyes were taken into consideration. It may be argued that this difference between groups is due to the procedure itself and the required postsurgical manipulations and due to the minimal invasiveness of the XEN procedure. In contrast to the data of the presented study, other groups have already demonstrated an improvement of mean BCVA in eyes after XEN implantation when combined with simultaneous phacoemulsification and lens implantation^33^.

In our cohort of eyes analyzed the frequency of necessary postoperative needling procedures was higher in the XEN than in the TET group (42% vs. 22%). Reported needling rates after XEN microstent implantation differ between study populations. Reported needling rates lie between 22 and 51% during the first year after surgery, and are even reported to be as high as 55% after 3 years of follow-up after XEN microstent implantation^14,18,27,28,34–39^. Length of follow-up, aggressiveness of postsurgical treatment, patient´s preferences and targeted IOP are among the numerous factors influencing needling rates. Our data point out that postoperative needling is more frequent after XEN microstent implantation than after TET, which has also been demonstrated before by Schlenker et al. This is a disadvantage of the procedure and has to be communicated with the patient prior to surgery.

It is known that TET is accompanied by a certain risk for postsurgical complications and therefore requires intensive postsurgical care^32,40,41^. According to Jünemann et al. the increased outflow of aqueous humor after TET leads to an increased conjunctival fibrosis and may lead to maculopathy, choroidal effusion, and suprachoroidal hemorrhage in hypotonic states^32^. In our two groups of eyes treated with either TET or XEN microstent implantation we found almost twice as many postsurgical complications in the TET group requiring more surgical attention than eyes in the XEN group. However, when appropriately treated occurring complications should be no hindrance in turning the final outcomes into an overall and long-lasting clinical success^32^. Nevertheless, invasive surgery puts additional burden on patients and therefore less invasive techniques are sought for^42,43^. The XEN microstent might be one viable alternative, despite the lesser IOP reducing and medication-lowering effectiveness in comparison to the gold standard TET. Our hereby presented data support this assumption. Patients in the TET group were more often affected by complications such as bleedings into the anterior chamber, choroidal detachment, hypotony, corneal erosion, and retinal detachment than patients in the XEN group. The APEX study also showed that the XEN implant’s safety profile compared favorably with that published for TET and glaucoma tube shunts^27^. Likewise, with the higher complication and postsurgical care rate in TET than in XEN group this present study demonstrates the XEN microstent’s more favorable safety profile. Picht et al. reported that about 50% of TET patients required postoperative additional therapeutic measures, specifically if scarring of the filtering bleb had been involved^44^. Picht et al. concluded that an intensive follow-up after TET was mandatory to avoid postoperative failure and that taking care of these factors after filtering surgery would increase success rates^44^.

Glaucoma usually occurs with increasing age and may be accompanied by additional age-related comorbidities with impact on the severity of the intraocular pressure and the patient’s adherence to medical treatment^45,46^. Given the different effectiveness of TET and XEN after 12 months, the decision which procedure to choose should be made with regard to the individual patient’s needs. Both procedures have pros and cons, as shown by this study. Smith et al. already emphasized that patients should be warned about the probability of necessary postoperative bleb interventions and application of topical IOP-lowering medication 12 months after XEN implantation^47^. Rooney et al. reported several cases of postoperative complications after XEN implantation. Gabbay et al. also stated that patients should be advised regarding failure rates, possible need for bleb revision and the resumption of medication use after XEN implantation^16,48^.

For this study cases of XEN micorstent implantation combined with phacoemulsification and IOL implantation and stand-alone XEN microstent implantations have been included. The fact that both groups of cases were included might pose a bias on the comparison to TET since no cases of combined TET and phacoemulsification were included. Reitsamer et al. already reported that postsurgical results concerning IOP and necessary IOP-lowering medication did not differ between combined and stand-alone XEN cases. However, Gillmann and colleagues reported that combined XEN implantation and phacoemulsification surgery had an increased risk for failure in POAG eyes compared to stand-alone XEN microstent implantation regarding their 3-year follow-up results^39^. Further subgroup analysis of our cases did not reveal differences of statistical significance concerning postoperative IOP development or necessary IOP-lowering medication in the combined or stand-alone XEN cases so far. Re-analysis of our subgroups after longer follow-up times would be desirable. Differences were only found between both XEN subgroups with respect to BCVA development. Combined cases showed a distinct improvement of BCVA, whereas BCVA remained mostly unchanged in the XEN stand-alone cases.

Another limitation concerning the utilized study design was the retrospective nature and the mode of patient collection with a previous setting of number of eyes included. A prospective study design would have made it possible to collect more reliable data. Due to the retrospective nature of this study IOP measurements might be biased considering inter-observer and inter-examination variability. IOP measurements were not performed in a blinded fashion or repeated multiple times as would be desirable. However, comparison of two different study groups might counteract this bias. A double-blinded study design is hardly manageable since both procedures are easily distinguishable at the slit lamp. However, a prospective study design with strict randomization would be desirable.

As another bias of the performed analysis, the percentage of eyes already undergoing IOP-lowering surgery before inclusion into this study could be seen. The fraction of eyes already undergoing glaucoma surgery were comparatively large with 41% and 44% in the TET and XEN groups. Nevertheless cases were added and analyzed because prior surgery comprised mostly non-incisional glaucoma surgery such as ALT and CPK, the performed procedure had to date back more than 1 year before XEN and TET surgery, IOP had to lie above targeted values under maximum tolerable therapy and the percentage of cases was comparable in both groups.

Nevertheless, concerning the hereby presented data both TET and XEN groups have proven to be significantly effective in lowering IOP and reducing dependence on IOP-lowering medication. Postsurgical complications were noted and taking corrective measures was necessary in both the TET and XEN groups. Finally, more long-term data for XEN implantations—beyond the 12- and 24-month mark—would be worthwhile.

## Data Availability

Datasets generated during the current study are available from the corresponding author on request.
